# Hand hygiene compliance and associated factor among nurses working in public hospitals of Hararghe zones, Oromia region, eastern Ethiopia

**DOI:** 10.3389/fpubh.2022.1032167

**Published:** 2022-12-07

**Authors:** Hamza Umar, Abraham Geremew, Teshager Worku Kassie, Gebisa Dirirsa, Kefelegn Bayu, Dechasa Adare Mengistu, Ashenafi Berhanu, Salie Mulat

**Affiliations:** ^1^Department of Nursing, Dire Dawa University, Dire Dawa, Ethiopia; ^2^Department of Environmental Health, College of Health and Medical Sciences, Haramaya University, Harar, Ethiopia; ^3^School of Nursing and Midwifery, College of Health and Medical Sciences, Haramaya University, Harar, Ethiopia

**Keywords:** hand hygiene, nurses, hospitals, infection prevention, health care workers

## Abstract

**Background:**

Healthcare workers are in constant contact with a wide variety of materials and surfaces, including waste, body fluids, mucous membranes, food, their own bodies, and the skin of patients. As a result, their hands are colonized by different groups of pathogens. Hand hygiene of healthcare workers is recognized to be the main factor in reducing healthcare-associated infections. Therefore, this study aimed to assess hand hygiene adherence and related factors among nurses working in public hospitals in eastern Ethiopia.

**Methods:**

An institutional based cross-sectional study was conducted in Hospital, Hararghe zone, Eastern Ethiopia from July 1 to 30, 2021. A total of 451 study participants were randomly selected, after the proportional allocation of study participants to each selected hospital. The data was collected using self-administered questionnaire and observation checklist. SPSS version 26 was used to analyze the data. Bivariable and multivariable analysis were employed to assess the association between outcome and independent variables. Finally, a *p*-value of < 0.05 was used as a cutoff point for statistical significance.

**Results:**

Out of 436 eligible nurses, the overall hand hygiene compliance was 37.4% [95% CI (0.33, 0.42)]. The overall compliance among those working in medical, surgical, OR ward, OPD, Gynecology/obstetrics, emergency ward, Intensive care units, Pediatrics, and other wards/departments was 46.8, 44.8, 35.7, 28.2, 20.7, 45.1, 23.1, 40.5, and 29.4%, respectively. The mean knowledge score was 21.6% (SD: 2.08). Furthermore, there was a statistically significant association between hand hygiene compliance and gender, work experience, training in hand hygiene, availability of running water, and knowledge of hand hygiene.

**Conclusion:**

The current study found overall compliance with hand hygiene accounted for 34.7%. Therefore, an exemplary worker may initiate others to do so, and strong managerial and leadership commitment may also help the workers stick to the rules and regulations to follow the multimodal hand hygiene practice as per WHO recommendation.

## Introduction

Hand hygiene is recognized to be the main factor in reducing health care-associated infections (HCAIs). Centers for Disease Control and Prevention (CDC) reported that healthcare workers do not wash their hands as often as possible, which contributes to the increasing number of HCAIs (1). According to the WHO, because healthcare workers constantly contact numerous substances and surfaces such as trash, bodily fluids, mucous membranes, food, their own bodies, and patients' skin, their hands become colonized by various bacteria. There are reports that 7 and 10% of hospitalized patients acquire at least one health-care associated infection in developed countries and developing countries, respectively (2–4). The Code of Standards and Conduct of the Nursing and Midwifery Council demands nurses and midwives to maintain a high level of practice and care at all times (5).

Healthcare Associated Infections are continuing to be a public health problem across the world because they are significantly associated with increased risks of mortality and morbidity (4). At least 1.7 million patients in the United States acquired HCAIs in 2017 alone, and around 99,000 of them died (4). Studies reveal that improving hand hygiene reduces HCAIs and Methicillin-resistant *Staphylococcus aureus* (MRSA) transmission rates (6, 7).

Improper hand hygiene is one of the leading causes of health care-associated infections. Compliance to hand hygiene among health care workers (HCW) in general is unacceptably low, especially in developing countries. Nosocomial infections, for example, are a major source of morbidity, death, and health-care expenditures among hospitalized patients globally (8, 9).

Furthermore, HCAIs cause prolonged hospital stays, increased microorganisms' resistance to antimicrobials, high costs, and increased mortality. Hand hygiene plays a great role in preventing HCAIs. There are different factors that hinder effective hand hygiene practice, including using gloves, inaccessibility and unavailability of alcohol-based hand rub, and inadequate water supply (4, 10–12). Moreover, lack of understanding of good hand hygiene practice, inadequate staff, overcrowding, and a lack of access to hand washing facilities, have been cited as contributing factors to poor compliance with hand hygiene among healthcare workers in developing countries (13, 14).

In Ethiopia, a few studies, conducted in central and northern parts, show a very low compliance rate for good hand hygiene by healthcare providers compared to the WHO 5 min for hand hygiene (12–18). Evidence on the hygiene practices of nurses in rural areas of the eastern part is limited. Therefore, this study was aimed to assess hand hygiene compliance and associated factors among nurses working in public hospitals in Eastern Ethiopia.

## Materials and methods

### Study setting and period

The Hararghe zone is located in the eastern part of Ethiopia in the Oromia regional state. The zone has seven public hospitals (four general hospitals and three primary hospitals) and 121 health centers. All hospitals provide inpatient, outpatient, emergence, and delivery services (19). The study was conducted from July 1 to 30, 2021.

### Study design

An institution-based cross-sectional study was conducted to assess hand hygiene compliance and associated factors among nurses working in public hospitals in the Hararghe Zones.

### Source and study population

All nurses working in public hospitals in the Hararghe zones were considered the source population. All randomly selected licensed nurses who had more than 6 months of working experience and are currently working in selected public hospitals were considered as the study population.

### Inclusion and exclusion criteria

This study included all nurses who had worked for at least 6 months at public hospitals in the Hararghe Zones. Nurses who were absent during the data collection for various reasons were excluded.

### Sample size determination

The sample size (n) was computed using the double population proportion formula using Epi-info version 7.01. The sample size calculation was based on a prior study that showed the availability of hand hygiene guidelines in the health care facility has an association with hand hygiene compliance (18), 95% confidence level, power of 80%, a 1:1 ratio of having hand hygiene guidelines among compliant and non-compliant nurses, and a 10% non-response rate. The final sample size estimated for this study was 451.

### Sampling method and procedure

**Figure 1:**
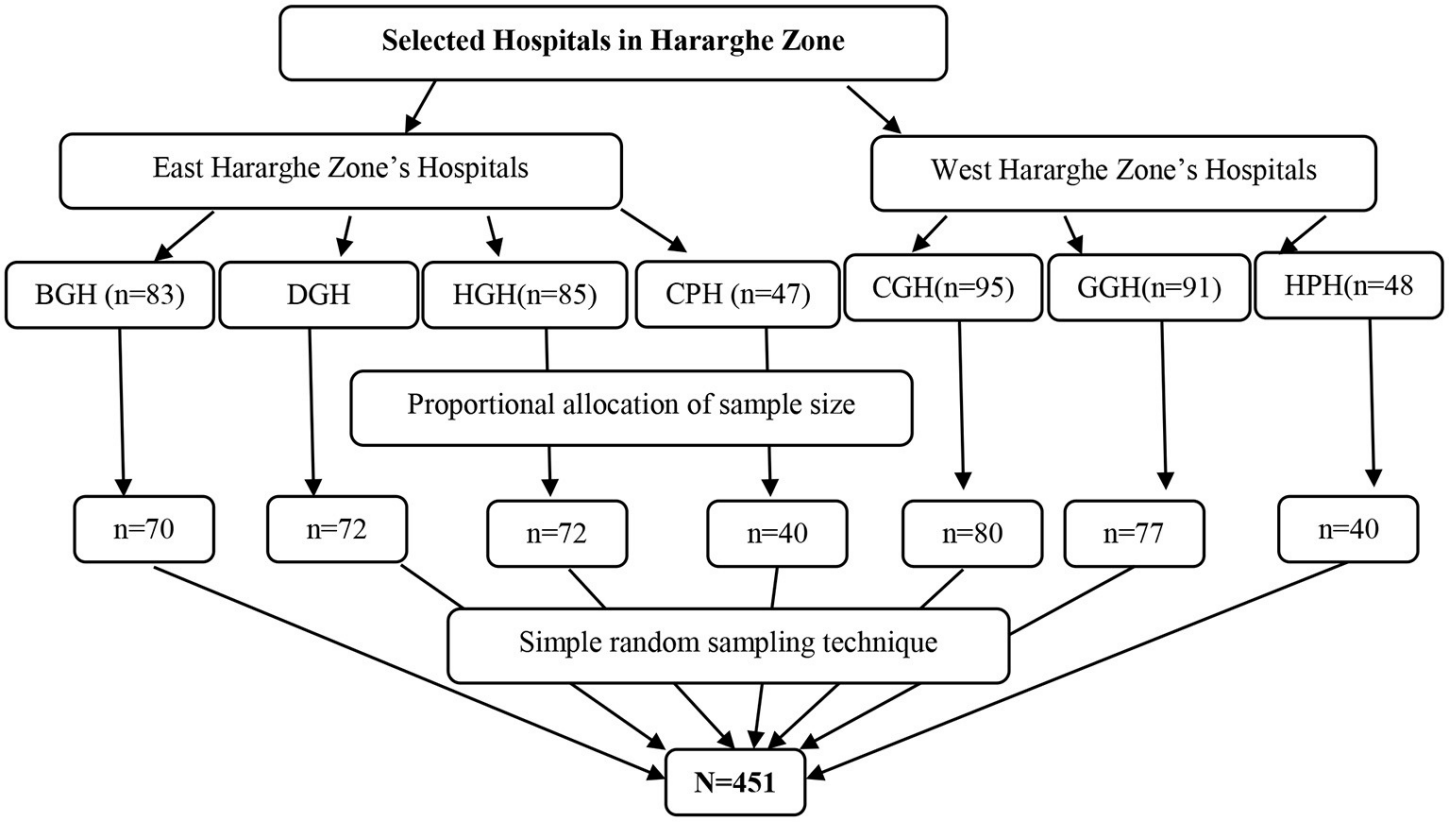
Sampling techniques used to select study participants for the current study, 2021. BGH, Bisidimo General Hospitals; DGH, Deder General Hospitals; HGH, Haramaya General Hospitals; CPH, Chelenko Primary Hospitals; CGH, Chiro General Hospitals; GGH, Gelemso General Hospitals; HPH, Hirna Primary Hospitals; N, Total sample size.

All seven public hospitals in the Eastern and Western Zones were considered for the current study. In the current study five general hospitals (Bisidimo, Deder, Haramaya, Chiro and Gelemso general hospitals) and two primary hospitals *(*Chelenko and Hirna Primary Hospitals) were included. The sample size distribution for each hospital was based on the proportion of nurses on duty at the time of data collection. Finally, a random sampling technique was used to select study participants from each facility (Figure 1).

### Data collection tools and procedure

The WHO hand hygiene compliance assessment questionnaire (20) was used to develop the standard self-administered questionnaire and observational checklist. The questionnaire was customized and developed to assess current hand hygiene compliance and associated factors. The tools include hand hygiene knowledge (12 items), administrative characteristics (eight items), the availability of a hand hygiene facility (nine items) *via* yes/no, nurses' hand hygiene practices *via* 5-point Likert scales (14 items), and ticking off what they do. It was prepared in the English language. Three data collectors and one supervisor were recruited and trained on data collection tools, consent, and ethical issues during data collection.

### Data quality control

The questionnaire was pretested on 5% of the participants outside the study area or in included hospitals to check its clarity, sequence, applicability, and validity. Then, the questions found unclear was modified based on the response provided during pretest before data collection. Each day, the completeness and consistency of the questionnaires were checked to ensure the quality of the collected data. Finally, the data were cross-checked using double data entry.

### Data processing and analysis

EPI-info version 7.2.4.0 was used to enter and clean data, which was then exported to SPSS version 26.0 for analysis. Frequencies and percentages of different variables were computed. To investigate variables associated with nurse compliance, a binary logistic regression model analysis was performed, and the goodness of fit for the fitted model was evaluated using the Hosmer-Lemeshow test. An odds ratio with a 95% confidence interval was computed to assess the presence and degree of association between the dependent and independent variables. In the binary analysis, a variable with a *p*-value < 0.25 was a candidate for multivariable analysis. A *p*-value of < 0.05 in multivariable analysis was used to declare a statistically significant association.

## Results

### Socio-demographic characteristics

The number of nurses included in the study was 436, with a response rate of 97%. The mean age of participants was 27 ± 4.5 SD years. Most of them were male (68.5%), had <5 years of experience (72.2%), and 56.7% of participants were married (Table 1).

**Table 1:** Socio-demographic characteristics of the study participants in Hararghe zones public hospitals, eastern Ethiopia, 2021.

**Variables**	**Category**	**Frequency**	**Percent**
Sex	Male	298	68.5
	Female	137	31.5
Age	18-24	89	20.4
	25-34	323	74.1
	> 35	24	5.5
Marital status	Single	185	42.4
	Married	247	56.7
	Divorced	3	0.70
	Widowed	1	0.20
Educational level	Diploma	140	32.1
	Degree	284	65.1
	Master and above	12	2.8
Working department/ ward/unit	Medical	62	14.2
	Surgical	58	13.3
	OR	42	9.6
	OPD	78	17.9
	Gynecology/obstetrics	29	6.7
	Emergency	82	18.8
	ICU	26	6.0
	Pediatrics	42	9.6
	Others	17	3.9

ICU, Intensive Care Unit; OR, Operation room; OPD, Out Patient Department.

### Hand hygiene compliance

**Figure 2:**
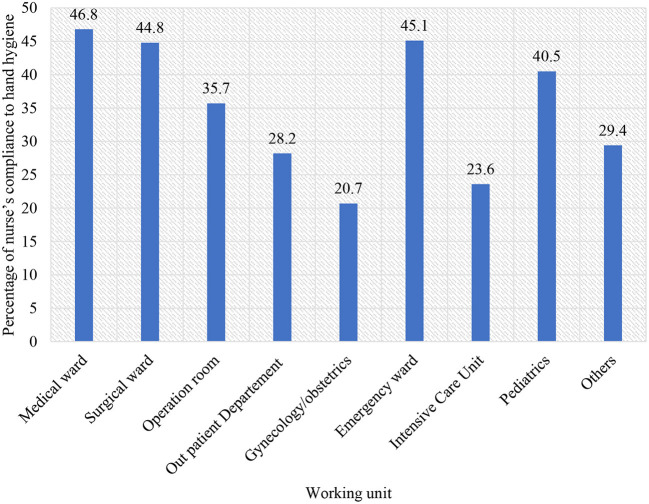
Hand hygiene compliance among nurses based on the working unit in Hararghe zones public hospitals, Eastern Ethiopia, 2021.

Overall hand hygiene compliance was 37.4% [163/436, 95% CI (0.33, 0.42)] among nurses working in the hospitals. The overall hand hygiene compliance with respect to the current working ward/department was as follows: the overall compliance of working in a medical ward was 46.8%, surgical ward was 44.8%, Operation Room (OR) was 35.7%, Out Patient Department (OPD) was 28.2%, gynecology/obstetrics ward was 20.7%, emergency ward was 45.1%, Intensive Care Unit (ICU) department was 23.1%, Pediatrics department was 40.5%, and nurses working in another ward/department was 29.4% (Figure 2).

### Knowledge about hand hygiene among nurses

The mean knowledge score was 21.6% (SD: 2.08). Among the participants, 67% [*n* = 294, 95% CI (0.63, 0.72)] of the participants scored 21.6% or more and were considered to have good knowledge. The most effective action for preventing nosocomial infections is proper hand washing. As a result, we asked nurses for alcohol-based hand rub preference when hands are visibly soiled and duration of hand rub knowledge. The correct answer that alcohol-based hand rubs should not be used when hands are visibly soiled and effective if applied for <60 s was known by 66.7 and 67.9% of nurses, respectively (Table 2).

**Table 2:** Nurses' knowledge of hand hygiene in Hararghe zones public hospitals in Eastern Ethiopia, 2021.

**Variable**	**Category**	**Frequency**	**Percent**
A-BHR should not be used when hands are visibly soiled.	Yes	291	66.7
	No	115	26.4
	I don't know	30	6.9
A-BHR will still be effective if applied for less than 60 s.	Yes	296	67.9
	No	95	21.8
	I don't know	45	10.3
Hand hygiene is required following the removal of gloves after patient contact.	Yes	355	81.4
	No	74	17.0
	I don't know	7	1.6
Single-use cloth towels and paper towels are acceptable for drying hands in patient care areas.	Yes	249	57.1
	No	161	36.9
	I don't know	26	6.0
Hand hygiene must be performed before contact with patient, following emptying of a drainage reservoir, and prior to and following venipuncture.	Yes	370	84.9
	No	57	13.1
	I don't know	9	2.1
When using an A-BHR to decontaminate hands they should be rubbed together until dry.	Yes	366	83.9
	No	42	9.6
	I don't know	28	6.4
Handling of paperwork is not one of the recommended situations for performing hand hygiene	Yes	247	56.7
	No	158	36.2
	I don't know	31	7.1
Hand hygiene is required following contact with the bed linen of a patient with MRSA.	Yes	350	80.3
	No	64	14.7
	I don't know	22	5.0
Hand creams and lotions are recommended for health care workers ‘hands.	Yes	291	66.7
	No	125	28.7
	I don't know	20	4.6
Gloves should not be reused when caring for different patients.	Yes	338	77.5
	No	81	18.6
	I don't know	17	3.9
The average cost of a hospital acquired infection in developed countries is approximately $10,000	Yes	199	45.6
	No	116	26.6
	I don't know	121	27.8
Approximately 20% of intensive care patients develop hospital acquired infection in developed countries	Yes	223	51.1
	No	87	20.0
	I don't know	126	28.9

A-BHR, Alcohol based hand rub; MRSA, Microbial Resistant Staphylococcus Aurous.

### Hand hygiene practices among nurses based on “my five moments of hand hygiene”

Regarding hand hygiene practices among HCWs (nurses), based on the “my five moments of hand hygiene” approach of the WHO, revealed that 66.3, 93.6, and 71.6 of nurses performed hand hygiene before aseptic procedure, after body fluid exposure, and after touching patient surroundings, respectively (Table 3).

**Table 3:** Nurses' hand hygiene practice based on World Health Organization “My five moments of hand hygiene” in public hospitals in Hararghe zones, Eastern Ethiopia, 2021.

**“My five moments of hand hygiene” indicators**	**Hand hygiene performed (%)**	**Compliance (%) (37.4%)**	**X^2^ (*p*-value)**
Before aseptic procedure	289 (66.3)	49.8	< 0.001
Before touching patients	132 (30.3)	81.1	
After body fluid exposure	408 (93.6)	38.2	
After touching patient	197 (45.2)	67.5	
After touching patient surroundings	312 (71.6)	51.6	

### Factors associated with nurses' compliance with hand hygiene

The bivariate analysis showed that having a sufficient budget for infection prevention in the hospital, regular feedback on hygiene performance, administrative motivations for hand hygiene practice, the presence of monitoring and evaluation in hand hygiene compliance, the availability of water in the working department/ward, the presence of ABHR for workers, the presence of the HH guide line, and knowledge of hand hygiene were all found to be significantly associated with hand hygiene compliance of nurses. The multivariable analysis showed that male nurse professionals' compliance with hand hygiene was 40% more likely compared to female nurse professionals (AOR: 0.60, 95% CI: 0.37, 0.97). The odds of nurses with work experience of 6–10 years complying with hand hygiene are 1.71 higher compared to those who have had <5 years of work experience (AOR: 1.71, 95% CI: 1.03, 2.86) (Table 4).

**Table 4:** Bivariate and multivariable logistic regression on factors associated with hand hygiene compliance among Nurses in Hararghe Zones public hospitals 2021.

**Variables**	**Hand hygiene compliance**			**COR (95% CI)**	**AOR (95% CI)**
	**Category**	**Good**	**Poor**		
Sex	Male	117	181	Ref.	Ref.
	Female	46	91	0.78 (0.51, 1.19)	0.60 (0.37, 0.97)^*^
Educational status	Diploma	52	88	Ref.	Ref.
	Degree	106	178	1.01 (0.66, 1.53)	0.91 (0.57, 1.47)
	Masters and above	5	7	1.21 (0.36, 4.00)	0.92 (0.22, 3.92)
Work experience	< 5 years	110	205	Ref.	Ref.
	6-10years	47	57	1.54 (0.98, 2.41)	1.71 (1.03, 2.86)^*^
	11–15 years	3	7	0.79 (0.20, 3.15)	0.54 (0.12, 2.39)
	>16	3	4	1.39 (0.31, 6.36)	1.53 (0.25, 9.50)
Hospital has enough budget for infection prevention activities	No	47	116	Ref.	Ref.
	Yes	116	157	1.83(1.20, 2.76)^*^	1.23 (0.75, 2.01)
Trained on hand hygiene	No	89	152	Ref.	Ref.
	Yes	74	121	0.92 (0.62, 1.35)	0.45 (0.26, 0.77)^*^
Presence of infection prevention committee in hospital	No	69	160	Ref.	Ref.
	Yes	94	113	0.96 (0.65,1.41)	1.12 (0.66, 1.87)
Regular feedback on hygiene performance from close supervisors	No	85	174	Ref.	Ref.
	Yes	78	99	1.61 (1.09, 2.39)^*^	1.25 (0.73, 2.12)
Any motivations of hand hygiene practice in hospital	No	72	176	Ref.	Ref.
	Yes	91	97	2.29 (1.54, 3.41)^**^	1.54 (0.92, 2,58)
Presence of monitoring and evaluation of hand hygiene compliance in hospital	No	77	172	Ref.	Ref.
	Yes	86	101	1.90 (1.28, 2.82)^**^	1.39 (0.80, 2.41)
Availability of running water in working department/ward	No	38	140	Ref.	Ref.
	Yes	125	133	0.29 (0.19, 0.45)^**^	2.19 (1.30, 3.71)^*^
Presence of alcohol hand rub for workers	No	28	107	Ref.	Ref.
	Yes	135	166	3.11 (1.94, 4.99)^**^	1.70 (0.96, 3.02)
Presence of a functional sink with soap for hand washing	No	47	147	Ref.	Ref.
	Yes	116	126	2.89 (1.90, 4.36)^*^	1.37 (0.83, 2.28)
Presence of posters for hand hygiene	No	41	101	Ref.	Ref.
	Yes	122	172	1.75 (1.14, 2.69)^*^	1.32 (0.79, 2.19)
Presence of hand washing guide line	No	53	144	Ref.	Ref.
	Yes	110	129	2.32 (1.55, 3.47)^**^	1.44 (0.89, 2.33)
Knowledge of hand hygiene	Poor	34	108	Ref.	Ref.
	Good	129	165	2.48 (1.58, 3.89)^**^	2.25 (1.35, 3.73)^*^
Attitude toward hand hygiene	Negative	70	126	Ref.	Ref.
	Positive	93	147	1.14 (0.77, 1.68)	0.82 (0.51, 1.30)

* *p* <0.05,

** *p* < 0.001.

## Discussion

This study reveals that the overall hand hygiene compliance of nurses in the public hospitals of the Hararghe zones was low at 37.4% (95% CI: 0.33, 0.42). The main factors associated with hand hygiene compliance of nurses were gender, work experience, training in hand hygiene, availability of running water, and knowledge of hand hygiene. Professionals' compliance with hand hygiene is low compared to the 50% compliance as per WHO recommendation (21).

Compliance was low across all working wards/departments, with the surgical ward having the highest compliance (46.8%). The low level of hand hygiene compliance in this study implicates the poor implementation of WHO's multimodal hand hygiene improvement strategies in hospitals. This could directly be related to the lack of hand hygiene promotion and monitoring, low hand hygiene training opportunities, and unavailability of hand washing facilities and materials at the point of patient care (15, 16, 22).

The finding of this study on hand hygiene compliance is higher with studies in Gondar University hospital (16.5%) (15), Gondar public primary hospitals (14.9%) (13), Dessie referral Hospital (17.6%) (22), Debre Birhan referral hospital (22.0%) (17), Wachemo University teaching hospital, in which hand hygiene compliance was reported at 9.2% (23) and a study conducted in Nigeria (31%) (24). In contrast, it was lower than previous findings from a general hospital in Addis Ababa (50.4%) (18). Indeed, the finding of this study is lower than a prior study conducted at a self-reported referral hospital in Kenya (80%). The possible assumptions for the disparity might be due to differences in hospital setting and capacity, availability and accessibility of hand washing facilities, and nurse's awareness and acceptance level.

Regarding the relation between gender and self-reported hand hygiene practices, this study reveals female nurses to have significantly lower hand hygiene practices than male nurses. These results are supported by previous studies in accordance with other studies, which also showed similar significant differences in the performance of hand hygiene between males and females (10).

In the current study, the results did not reveal any kind of association between hand hygiene practice and the educational level of participants. In contrast, a prior study showed there is a significant association between educational level and hand hygiene practice (18).

In this study, work experience was one of the factors that had a significant association with hand hygiene compliance of nurses. Those who had 6–10 years of working experience had 1.71 times more hand hygiene compliance than those who had ≤ 5 years of experience. Having training on hand hygiene was also one of the factors that was significantly associated with the compliance. The result reveals that those who were trained on hand hygiene had 0.45 times more practice on hand hygiene compliance than those who did not have training. This finding is in line with prior studies conducted in different hospitals (13, 15, 18), and Nigeria (24).

In this study, 67% of participants had good knowledge of hand hygiene. The result shows that in the current study, participants show a higher knowledge level on hand hygiene than a study conducted in Meshhad, Iran in which only 10.6% of participants and 10.9% of nurses scored good knowledge (25). The possible assumption for the disparity might be the higher percentage of participants (53.4%) in the prior study who had been trained on hand hygiene than the participants in the current study (44, 7%). This implies that having training may improve the knowledge of healthcare providers on hand hygiene and, as a result of improved knowledge, healthcare providers become more compliant toward the gold standards of WHO recommendation on hand hygiene (10, 18).

Knowledge of nurses are found positively associated with compliance. Consequently, nurses who had a good knowledge on hand hygiene were 2.25 times more compliant than those who had poor knowledge. This result is supported by a study conducted in Gondar primary hospital and in Kuwait (13, 26) in which healthcare workers who had good knowledge were more compliant with hand hygiene than those who had poor knowledge. Therefore, addressing healthcare providers knowledge about hand hygiene, as well as strategies for cognitive, emotional, and behavioral approaches, such as patient involvement in hand hygiene intervention by applying the multimodal training program are important to improve the level of compliance (25).

This study shows that the availability of running water in the working department/ward was also significantly associated with hand hygiene compliance. The nurses who were working in wards/departments where running water is available had 2.19 more compliance than their counterparts.

In the current study, compliance with hand hygiene was in line with the level suggested by WHO's indications (21, 27), except for hand hygiene after touching body fluid. Consistent to WHO's indication, higher levels of compliance were observed before touching a patient, before an aseptic procedure, after touching a patient, and after touching patient surroundings. On the other hand, compliance with hand hygiene was relatively lower after body fluid exposure. This finding implies that, while healthcare providers were protected from healthcare-associated infections for themselves and their patients, they were at a higher risk of infection than patients. The possible assumption for this exposure might be the reason for low hand hygiene compliance. Therefore, it is recommended to strictly adhere to hand hygiene standards to prevent the spread of infections (28).

## Limitations

The main limitation of this study is that the results are based on a self-reported questionnaire. There could be an overestimation of hand hygiene compliance. As a result, an observation-based assessment of hand hygiene practice to hand hygiene compliance can determine the extent to which professionals reported and observed practices differ. Another limitation of the study is that no proxy measures like hospital-acquired infection rates of different departments or access to hand hygiene facilities of nurses in different departments were not assessed in the study to validate the self-reported hand hygiene practice. The study also acknowledged biases from the study design and nature of participants, like fewer older age (≥ 35) study participants.

## Conclusion

This study was conducted to assess the self-reported practices of hand hygiene among nurses working in public hospitals in the Hararghe Zones, Oromia Region, Ethiopia. The current study depicts overall compliance of hand hygiene of 34.7% and, as per the WHO “five moments of hand hygiene” approach, 66.3, 93.6, and 71.6% of nurses performed hand hygiene before aseptic procedure, after body fluid exposure, and after touching patient surroundings, respectively.

Female nurses, those with limited work experience, and those who did not receive hand hygiene training require on-the-job training and educational intervention to improve their compliance with hand hygiene practices and knowledge of hand hygiene practices. Furthermore, motivating exemplary workers may encourage others to do the same, and strong managerial and leadership commitment may also help workers adhere to the rules and regulations to follow the WHO recommendation for multimodal hand hygiene practice.

## Data availability statement

The raw data supporting the conclusions of this article will be made available by the authors, without undue reservation.

## Ethics statement

The studies involving human participants were reviewed and approved by the Institutional Health Research Ethics Review Committee of the College of Health and Medical Sciences, Haramaya University. The patients/participants provided their written informed consent to participate in this study.

## Author contributions

HU conceived the idea, collected the data, and played a major role in this research. HU, AG, TW, GD, KB, DM, AB, and SM contributed to data analysis, writing, and editing the document. HU, AG, DM, and GD gave valuable ideas for the manuscript and revised it. All the authors read and approved the final version to be published and agreed on all aspects of this work.
